# WaspAtlas: a *Nasonia vitripennis* gene database and analysis platform

**DOI:** 10.1093/database/bav103

**Published:** 2015-10-09

**Authors:** Nathaniel J. Davies, Eran Tauber

**Affiliations:** Department of Genetics, University of Leicester, University Road, Leicester LE1 7RH, UK

## Abstract

*Nasonia vitripennis* is a parasitoid wasp which is becoming an important model organism for parasitism, epigenetics, evolutionary and developmental genetics. WaspAtlas is a new gene database in which we have compiled annotation data from all available *N. vitripennis* releases along with a wealth of transcriptomic data, methylation data and original analyses and annotations to form a comprehensive resource to aid the study of *Nasonia*. WaspAtlas allows users to explore gene structure and function, to compare expression data across sexes, tissues, developmental stages and conditions, and to explore published data relating to gene(s) of interest. WaspAtlas is easy to navigate and the database is easily searchable through the web interface. Detailed illustrations are provided for splice variants, protein domain predictions and the results of analyses. The website also functions as an analysis platform analysis for *Nasonia*, providing a set of tools designed to perform common analyses including GO term overrepresentation and RNAi off-target prediction. WaspAtlas will act as a hub for published data relating to *Nasonia* genes, and will be continually updated with new data to reflect the state of *Nasonia*-omics research.

**Database URL**: http://waspatlas.com

## Introduction

Parasitoids are an extremely diverse group of insects, making up roughly 10% of all described insects (1). The largest group of parasitoids in the insect kingdom, and thus an important cornerstone of animal biodiversity, is the parasitoid wasps, whose life cycle involves acquisition of nutrients from a host organism. The great diversity of parasitoid wasps is matched by a great diversity of hosts, many of which are pest species. Parasitoid wasps are thus widely used commercially in environmental friendly biological pest control (2).

*Nasonia vitripennis* is one such parasitoid wasp, and is a generalist able to parasitize a wide variety of flies including the blowfly (3), a common pest. *Nasonia* is an important model for parasitism, and has been used extensively in studies on host–parasitoid relationships, e.g. (4–7). *Nasonia* is also becoming an important insect model (8) in other areas, as an alternative to fruit fly *Drosophila*, as it offers several advantages over existing model systems. These advantages include a haplodiploid sex determination system (9), simple rearing, a fully functional DNA methylation system (10–12), robust circadian (13–15) and photoperiodic (16) responses, a fully sequenced genome (17) and a systemic RNAi response (18, 19). Also of note is *Nasonia*’s position in the insect order Hymenoptera, an order which evolves more slowly than the order to which *Drosophila* belongs, Diptera (20).

Since the original publication of the *Nasonia* genome (17), the assembly has been improved and detailed annotation projects are ongoing (Supplementary Note S1). The level of annotation between assemblies and annotations varies, e.g. the EvidentialGene dataset (21) (mapped to the first genome build) contains UTR annotation for 97% of gene models and has a significant amount of associated GO (gene ontology) annotation, whereas OGS v1.2 (mapped to the latest genome build, adopted by Ensembl) only has UTR annotation for 37% of gene models and has relatively little gene ontology (GO) (22) annotation. User-interface aided access to the annotation files also varies significantly between projects, so trade-offs exist when selecting the appropriate reference annotation for a given project. Each gene annotation projects has its own set of gene identifiers, making comparisons between studies which use different reference annotations difficult, as there is no existing method for converting identifiers in batch.

Reflecting its position as an important model organism, several RNA-seq datasets have been recently produced for *Nasonia* and a few genome-wide methylation datasets also exist. Together, this bulk of data gives us gene expression information for both male and female wasps, various tissues, important developmental stages and different experimental conditions. Unfortunately, these datasets are currently scattered throughout different studies and has been mapped using various reference annotations. Given the number of experiments being carried out with *Nasonia*, it would be useful to have this data in one place to be able to find out where and when a particular gene is most highly expressed or to compare the expression patterns of groups of genes.

We here present a database combining data from all *Nasonia vitripennis* annotation projects, original annotation works and analyses, all currently available RNA-seq transcriptome libraries/microarray data and existing DNA methylation data. In WaspAtlas, each *Nasonia* gene is complete with, where possible, GO annotations, PFAM domain predictions (23), orthologs in other important model species, expression data comparing sexes, tissues, developmental stages, and experimental conditions and data from as yet unpublished analyses. To demonstrate the utility of our database, we perform an analysis of potential housekeeping genes using the data integrated into WaspAtlas, and provide lists of reference genes for use in qPCR or other applications. WaspAtlas will be continually updated with the latest annotation data, and new omic datasets will be integrated into the website as they are made available.

In summary, WaspAtlas provides the following features:

- All *Nasonia vitripennis* annotation releases
- RNA-seq, microarray and DNA methylation data
- Easy to navigate web interface, with illustrations
- Gene search functionality
- Genome browser
- Tools
  - GO term/PFAM domain hypergeometric overrepresen tation
  - RNAi off-target prediction
  - Batch gene information retrieval

## Data processing

In order to provide a complete annotation for *Nasonia* and to create a complete mapping between all gene annotations, gene models from four different annotation projects were intermapped using a combination of all extant gene equivalency mappings and collapsed into as few loci as possible (Supplementary Note S2). All transcripts from the gene models used were included, along with information on coding sequences, which were then translated to produce the protein sequences for each protein-coding transcript. The results of the interannotation mapping are available to be downloaded in batch from the *Downloads* page.

Once these more comprehensive loci had been built, we added GO-term associations from Nasoniabase (21) and ensembl (24). These GO terms were then expanded by traversing the GO annotation tree to include more general terms. To assign protein domains to amino acid sequences, we used HMMer (25) to predict PFAM domains for each amino acid sequence in WaspAtlas (Supplementary Note S3). In order to facilitate easy comparisons between *N. vitripennis* genes and those of other more well established model organisms we calculated orthologs from 11 different species (including human, mouse, *Caenorhabditis elegans* and *Drosophila melanogaster*) using a reciprocal best blast hit (RBH) (26) approach (Supplementary Note S4). These orthologs were supplemented with orthology data from Ensembl (24) where available.

RNA-seq libraries comprising 43 samples across 7 experiments (Supplementary Table S1) (11, 27–30) were mapped to NCBI Nvit 2.1, as the latest annotation, using the tophat 2 v2.1.0 (31) with novel junction discovery disabled. Cufflinks and cuffnorm were used with geometric (DEseq) normalization (32) within datasets and tissues to calculate the FPKM values for each transcript. For inclusion into WaspAtlas, the mean and standard deviation were calculated for each distinct tissue and dataset, and enrichment values calculated relative to 24-h whole body samples for each sex (30). Tiling microarray data showing gene expression during several crucial stages of female development (11) and tissues was also downloaded and integrated. In addition to these expression datasets, an RRBS methylation dataset showing the differences in methylation between female wasps exposed to long day photoperiods and those exposed to short day photoperiods (Pegoraro *et al.*, unpublished data) was integrated into the database, allowing users to compare expression data with genome-wide methylation data.

## Features and usage

**Figure 1.**
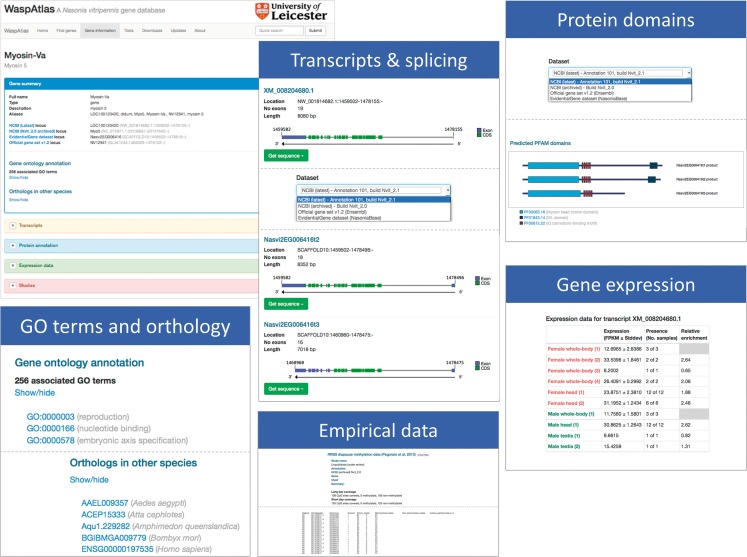
Overview of the gene information page showing the information available for each gene in WaspAtlas. The gene summary screen is shown on the top left. The various information sections are shown next to it.

Access to the WaspAtlas database is provided through a web-based interface. The interface can be conceptually divided into three main components: gene summaries, custom searches and tools. The gene summary page for each gene (Figure 1) is divided into five sections: (i) a brief summary describing the gene identifiers associated with this gene in different annotation releases and their locations on the various genome builds. Also detailed in this section are the annotated GO terms and orthology data, (ii) a transcripts section showing all annotation splice variants for the gene in question, containing detailed information, illustrations and downloadable sequences for all annotated transcripts, selectable by annotation release, (iii) a protein annotation section containing, for each splice variant, a schematic diagram of predicted PFAM protein domains and their locations within the protein, again selectable by annotation release, (iv) a gene expression section, showing the levels of gene expression in both sexes in various conditions and developmental stages, (v) a studies section, containing data from published studies relating to the gene being browsed.

**Figure 2.**
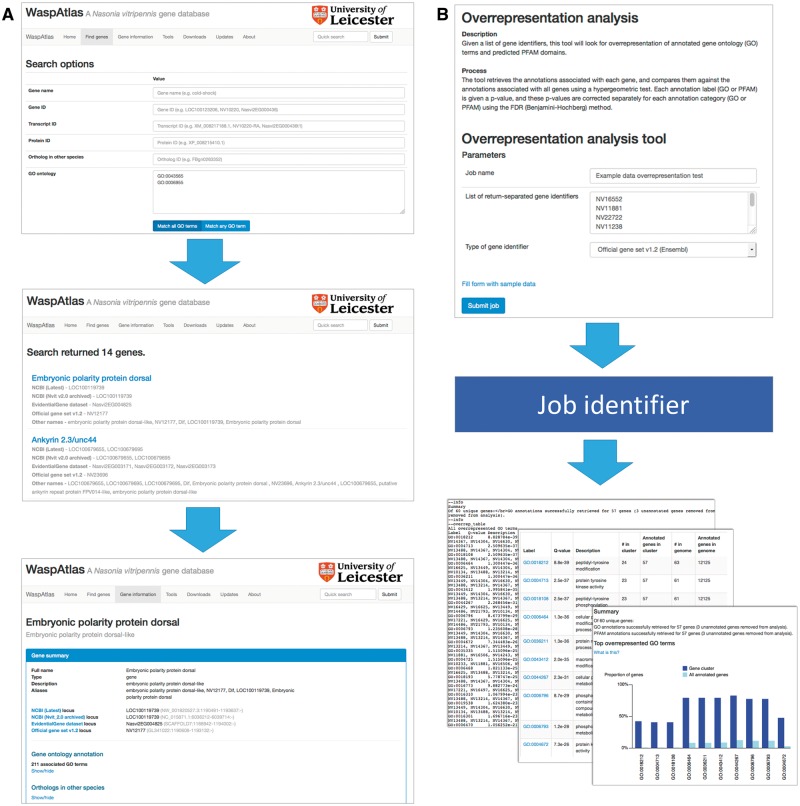
Analysis with WaspAtlas. (**A**) Use case of the advanced search function, performing a search for transcription factors involved in immune response. (**B**) Use case of the GO overrepresentation tool, showing input and output.

Searching for genes (Figure 2A) is straightforward, and can be carried out using the quick search or the advanced search. The quick search box in the upper right hand corner of every page will scan the database for genes with a certain name, symbol or identifier or genes annotated with a given GO term or PFAM domain. The advanced search gives users more control over their search terms, and allows users to search for groups of GO terms/PFAM domains, and gives a greater range of fields to search (e.g. by orthologous genes in other species). Genes can also be located using the genome browser, which is linked to and from the gene information page.

WaspAtlas also functions as an analysis platform for performing common analyses with *Nasonia* genes using the latest and most complete functional annotation available. Currently, users can perform GO term/PFAM domain overrepresentation tests using clusters of genes, predict potential RNAi off-targets for a given double-stranded RNA (dsRNA) fragment, and retrieve detailed functional annotation and intermapping for groups of genes at a time. To perform an analysis (Figure 2B), the user fills in the necessary parameters and is given a job identifier which can be used to track the job’s progress. Upon job completion, the user will be presented with a graphical and/or textual summary of the results along with a link to download the raw data. A detailed description of the methods used is shown alongside each tool, along with the option to test the tool with sample data.

## Transcriptome analysis

Using the RNA-seq data integrated into WaspAtlas, we performed an analysis of gene expression profiles and potential ‘house-keeping’ genes, with an aim to identify those genes which exhibit constant expression within, and perhaps even across, tissues. The genes identified from such an analysis would be suitable for use in normalization procedures (e.g. in qPCR).

To examine how similar gene expression profiles are between tissues, we calculated the mean expression values of each transcript in all samples within each dataset, doing this separately for sex (where both sexes were sequenced within a single experiment). We then calculated the correlation coefficient of the transcript expression values between all pairs of datasets and used these values to perform hierarchical clustering in R (33). The results of this analysis (Supplementary Table S2) show that different tissues appear to have very different gene expression profiles. Interestingly, although whole-body gene expression profiles differ extensively between males and females [Cor < 0.5, previously reported (30)], the male and female head transcription program is highly similar (Max cor > 0.84), which would perhaps enable direct comparisons between male and female heads in differential expression studies.

To look for potential house-keeping genes, we first found those genes with low variance within datasets. For each transcript in each dataset, we calculated the coefficient of variance of its expression across all samples. The transcripts with a coefficient of variance in the bottom 10% of values (i.e. the most stably expressed) and FPKM means ≥ 30 (i.e. expressed at a level high enough to be easily detected) were tested for overrepresented GO terms using the WaspAtlas overrepresentation tool. GO terms commonly significantly overrepresented (*q* < 0.01) in these house-keeping sets of genes included functions to do with ribosomes, organelles and mitochondria, suggesting a true fundamental housekeeping role for these genes. Although a few of these housekeeping genes were common between all datasets, the ratios between these genes were unstable, suggesting that there is no obvious set of housekeeping genes suitable for normalization across all tissues and conditions. Lists of all of these tissue-specific housekeeping genes are available to download from WaspAtlas for use in expression normalization.

## Implementation

WaspAtlas was implemented in Perl using the Catalyst development framework, and runs on an Apache server with a MySQL database. Template toolkit was used for frontend development, and all illustrations are drawn using JavaScript and HTML canvas. This research used the SPECTRE High Performance Computing Facility at the University of Leicester.

## Future development

WaspAtlas is the most comprehensive *Nasonia* resource developed to date, providing an easy to explore interface for accessing the most detailed *Nasonia* gene annotation available as well as the most current omic data produced by the *Nasonia* community. WaspAtlas also provides an analysis platform for working with this data, and allows the WaspAtlas data to be downloaded in batch for genome-wide analyses. WaspAtlas will be updated as more data is produced to ensure an up-to-date database of the current state of affairs of *Nasonia* research and more tools will be provided for working with this data. In the immediate future, we plan to integrate more methylome data and to expand the annotation of non-coding RNAs.

## Supplementary data

Supplementary data are available at *Database* Online.

## Funding

Midlands Integrative Biosciences Training Partnership (MIBTP) program. This work was funded by the Biotechnology & Biological Sciences Research Council (BBSRC), Grant BB/M01116X/1 for the Midlands Integrative Biosciences Training Partnership (MIBTP) and grant BB/K001922/1 to ET.

*Conflict of interest.* None declared.

## Supplementary Material

Supplementary Data
