# Supplementary material for: WaspAtlas: a Nasonia vitripennis gene database and analysis platform
Source: Database (Oxford). 2015 Oct 9;2015:bav103. doi: 10.1093/database/bav103 (PMC4599445; doi:10.1093/database/bav103)
Supplement: Supplementary Data [file supp_2015_bav103_index.html]

Supplementary Data 

# WaspAtlas: a *Nasonia vitripennis* gene database and analysis platform

## Supplementary Data

files

- Supplementary Data - xlsx file
- Supplementary Data - xlsx file
